# The acceptability of overdose alert and response technologies: introducing the TPOM-ODART framework

**DOI:** 10.1186/s12954-023-00763-4

**Published:** 2023-03-26

**Authors:** Josh Dumbrell, Hadi Daneshvar, Alberto Oteo, Alexander Baldacchino, Catriona Matheson

**Affiliations:** 1 Drugs Research Network Scotland, University of Stirling, Stirling, UK; 2 Faculty of Social Science, University of Stirling, Stirling, UK; 3 University of St Andrews Medical School, University of St Andrews, St Andrews, UK

**Keywords:** Opioids, People who use opioids (PWUO), Overdose (OD), Public health, Harm reduction, Overdose alert and response technologies (ODART), TPOM-ODART, Overdose digital technologies

## Abstract

**Background:**

Opioids were implicated in approximately 88,000 fatal overdoses (OD) globally. However, in principle all opioid OD are reversible with the timely administration of naloxone hydrochloride. Despite the widespread availability of naloxone among people who use opioids (PWUO), many who suffer fatal OD use alone, without others present to administer the reversal agent. Recognising this key aspect of the challenge calls for innovations, a number of technological approaches have emerged which aim to connect OD victims with naloxone. However, the acceptability of OD response technologies to PWUO is of key concern.

**Methods:**

Drawing on the Technology People Organisations Macroenvironment (TPOM) framework, this study sought to integrate acceptability-related findings in this space with primary research data from PWUO, affected family members and service providers to understand the factors involved in harm reduction technology acceptability.

A qualitative study using a focus group methodology was conducted. The participant groups were people with lived experience of problem opioid use, affected family members and service providers. Data analysis followed a multi-stage approach to thematic analysis and utilised both inductive and deductive methods.

**Results:**

Thirty individuals participated in one of six focus groups between November 2021 and September 2022. The analysis generated six major themes, three of which are reported in this article—selected for their close relevance to PWUO and their importance to developers of digital technologies for this group. ‘Trust—in technologies, systems and people’ was a major theme and was closely linked to data security, privacy and confidentiality. ‘Balancing harm reduction, safety and ambivalence’ reflects the delicate balance technological solutions must achieve to be acceptable to PWUO. Lastly, ‘readiness—a double bind’ encapsulates the perception shared across participant groups, that those at the highest risk, may be the least able to engage with interventions.

**Conclusion:**

Effective digital strategies to prevent fatal OD must be sensitive to the complex relationships between technological, social/human, organisational and wider macroenvironmental factors which can enable or impede intervention delivery. Trust, readiness and performance are central to technology acceptability for PWUO. An augmented TPOM was developed (the TPOM-ODART).

**Supplementary Information:**

The online version contains supplementary material available at 10.1186/s12954-023-00763-4.

## Introduction

**Fig. 1:**
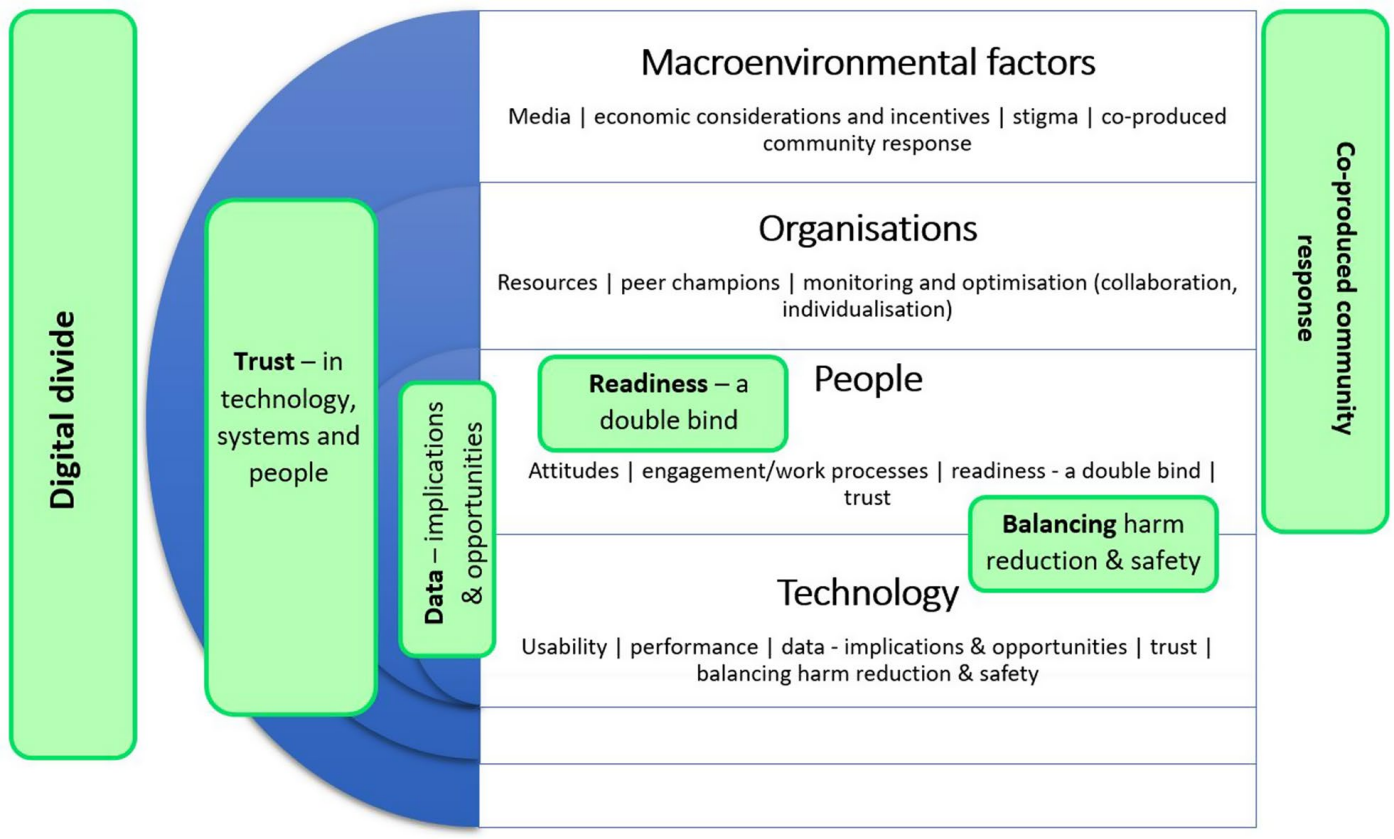
TPOM-ODART framework for PWUO

The most recently available global data indicate that there were 128,000 deaths directly related to drug use in 2019, with 88,000 of these being associated with illicit and prescribed opioid use [1]. North America and parts of Northern Europe are disproportionately burdened by opioid-related overdose (OD) deaths, together accounting for more than half of this figure [1]. Moreover, amid the COVID-19 pandemic, Canada saw a 98% rise in opioid-related OD deaths, while the USA exceeded 100,000 annual deaths for the first time [2]. At the same time, Scotland, in the UK, recorded a drug-related death (DRD) rate of 327 per million (aged 15–64) in 2020, which is more than twenty times the European average [3].

Health, social and structural factors such as long-term drug use, history of overdose, criminal justice involvement, poor physical and mental health and housing status contribute to the risk of fatal OD [4–6]. As these independent risk factors multiply and intersect, an individual’s likelihood of fatal OD [7] and premature mortality increases [8]. However, in addition to predicting DRD, this risk profile also indicates contact with support services capable of altering an individual’s trajectory through the provision of harm reduction resources and advice.

Fatal opioid OD are mostly preventable with the timely administration of naloxone—an opioid antagonist which temporarily reverses OD [9]. National naloxone distribution programmes have become key measures internationally in addressing fatal OD [10–12]. These programmes engage, educate, train and provide naloxone kits to communities of people who use opioids (PWUO), affected family members and service providers [13, 14]. Despite these measures, OD deaths have escalated dramatically, not least due to the propensity of individuals to use drugs alone [15–17]. This challenge calls for innovative solutions which can support the timely identification and early response to an OD.

The development of overdose alert and response technologies (ODART) follows shifts over the past decade towards digitally mediated support options [18, 19], while research in this area increased significantly during the COVID-19 pandemic, driven by restrictions on face-to-face service provision [20, 21]. Yet, the acceptability of such technologies to PWUO remains a key concern [22], anticipating a negative impact to the uptake of, and engagement in, these interventions.

Oteo et al. [19] offered an overview of different technologies developed internationally to address fatal overdose. In summary, their review identified [1] wearable devices and sonar-based room sensors which detect indices of overdose and broadcast alerts, and [2] smartphone applications which monitor drug use, provide OD prevention information and/or support the development of naloxone responder networks.

Across studies spanning the range of described technologies, Oteo et al’s review shows ‘trust’ to be a prerequisite for the acceptability and feasibility of ODART interventions [23–26]. Pooled findings indicate, for example, a preference among participants (PWUO) for trusted individuals, as opposed to emergency services, to be alerted to OD events, due largely to fears of discrimination and potential arrest from accompanying police. Also, impeding acceptability for participants in some studies was a shared sense of within-group distrust, stemming from the perception that ill-intentioned peers (i.e. PWUO) might steal from OD victims to whose vulnerability they were alerted [25, 27].

Oteo et al’s review also revealed high willingness among PWUO with access to smartphone technology towards wearing various ODART devices [23, 26, 27]. Factors independently associated with willingness included being in methadone treatment, having previous experience of overdose and a history of chronic pain. Negatively affecting ODART acceptability, by contrast, was a perceived aversion among PWUO towards ‘losing a high’ due to the effective administration of naloxone by responders. Two articles situated people experiencing homelessness/transient lifestyles and those with a low tolerance for opioids as having the most to gain from ODART interventions [26, 27]. Capturing greater detail on the scale of the challenge for ODART designers/implementers, however, Oteo et al’s review shows homelessness to be negatively associated with willingness to utilise (acceptability) and intervention feasibility [23, 26, 27]

The study aimed to gain an in-depth understanding of stakeholders’ views regarding the acceptability of ODART for PWUO in Scotland. Guiding this undertaking, the Technology, People, Organisations, Macroenvironment (TPOM) framework [28] was selected and adopted for its capacity to incorporate the multi-faceted individual, social and environmental perspectives associated with digitally mediated interventions. Originally designed to guide both formative and summative evaluations in health informatics, the TPOM offers a useful framework through which to explore the population/purpose specific factors involved in the acceptability of technology for reducing fatal OD. However, the unique complexity of the ODART environment provides a strong argument for adjusting and augmenting the framework to maximise the fit for ODART acceptability, thus addressing a secondary aim of the study.

## Methodology

### Study design and recruitment

A qualitative study was conducted between November 2021 and September 2022 using a focus group methodology. Target groups were people with lived experience (LE) of opioid use (current or recent illicit/prescribed) [*n* = 9], affected family members (FM) [*n* = 4] and service providers (SP) working alongside PWUO [*n* = 17].

Participants from each stakeholder group were recruited through the Drugs Research Network Scotland’s existing networks across frontline services and family support groups. Participation from frontline SP was supported via the DRNS newsletter (with a circulation of 400 subscribers) and social media. LE and FM participation was facilitated via direct approach by author JD to third sector services that support people with drug problems including via homeless services. A purposive sampling approach was applied, by which focus group participants were selected on the basis of their lived/professional experience as members of the target populations [29]. This approach permitted in-depth exploration [30] of ODART acceptability from the perspectives of stakeholders implicated in the effective delivery of digital harm reduction interventions in Scotland.

### Data collection

All data collection was undertaken through online focus groups. Six sessions were conducted in total with two for each target group. Focus groups ranged in duration from 70 to 90 minutes. The focus groups focused on participant views regarding presented technologies and their application to PWUO. The focus group format enabled participants to expand upon the views of others, as well as re-evaluate and reconsider their own understandings of specific experiences [31].

### Procedure

Participants were presented with examples (see Additional file 1: Appendix 1), including pictures and videos of ODART currently in use or as prototypes internationally, with participants invited to discuss each in turn.

### Theoretical framework

Several potential analytical frameworks were considered for guiding the development of a model of technology acceptability in the ODART space, including more typical harm reduction frameworks [32, 33], and those specifically focused on technology [34, 35]. However, the TPOM, constructed from a substantial dataset covering a range of care settings, has demonstrated its capacity for supporting formative assessments of health information technology implementation [28]. Thus, the model was expected to permit sufficient characterisation of the target population, while capturing the technological and feasibility-related areas associated with digital OD intervention delivery. The exact role of the framework in the creation and organisation of data is clarified immediately below.

### Analysis

Guided by Braun and Clarke’s [36] approach, a mixture of inductive and deductive thematic analysis was used. For example, though research questions were exploratory and experiential, and therefore suited to an inductive approach, theoretical constructs were consistently used to interpret data or to ‘render visible issues that participants did not explicitly articulate’ [37]. First, transcripts were read carefully twice with initial ideas being noted and revised iteratively. Second, line-by-line coding was undertaken using NVivo 12 [38] qualitative data analysis software. This followed an inductive process which involved grouping meaningful units of text into analytic categories under provisional labels. Third, codes were collated into potential themes based on semantic consistency. Fourth, the fit between provisional themes, their constituent codes and data extracts were continually reviewed. Fifth, the ongoing refinement of themes included examining their relations to each other, and the TPOM framework. This involved a process of deductive analysis in which the identified themes (or thematic descriptions) were transferred onto the TPOM (see Figure 1). In addition to demonstrating model fit, this deductive approach served two functions: it (1) allowed data to be classified in ways relevant to ODART designers/implementers and (2) facilitated the streamlining of participant findings. Sixth, in the final analytic stage, extracts were selected which exemplified the themes in relation to the TPOM. A selection of relevant outputs from this final analytic stage follows in the next section in which an augmented TPOM for this target group and purpose is presented. Our analysis and adoption of framework was informed theoretically by the socio-technical approach [39].

## Findings

All participants from each stakeholder group lived in Scotland. Three interrelated themes, derived from participant accounts, are reported below. Drawn from an original six key themes, the findings presented here serve to elucidate the depth and scope of the challenge to designers and implementers of digital OD prevention interventions. Though similarly compelling, the themes ‘*digital divide*’, ‘*data—implications and opportunities*’ and ‘*co-produced community response*’ were less distinctive to the population/intervention. The choice of themes, therefore, reflects a desire to establish the unique qualities of the population, and it is hoped to consider the model fit and support arguments for augmentation of the TPOM framework (see referent model in Additional file 2: Appendix 2).

### Trust—in technologies, systems and people

Spanning three dimensions of the TPOM, trust was a major theme relating to the acceptability of technological solutions for reducing OD. From a technological perspective, trust was closely linked to data security and permissions. Correspondingly, trust in service providers (i.e. those tasked with ensuring data protection) and potential OD responders was expected to determine ODART acceptability and use. Such trust-based conditions also exposed tensions in relation to the macro environment, namely between harm reduction, which normalises illicit drug use, and law enforcement.

Participants with experience of drug use felt that broad acceptance of technology would require assurances from developers and intervention delivery teams that end-user data would remain inaccessible to authorities.

“As long as there was an assurance that the app wasn’t going to trigger anything as far as law enforcement.” (LE)

Across groups, participants stressed the centrality of trusted, provider-led support, particularly for those at greatest risk, with providers best placed to advance such assurances.

“I suppose it’s also the case that the provider can convince a user that they’ve built a level of trust in the system themselves and assure them that way that it’s a trustworthy system.” (FM)

The social/human components associated with trust centred around who might best respond to a technology-detected OD. For example, concerns were raised across participant groups about the potential for responders who are actively using drugs to take advantage of OD victims.

“It’s just that a lot of drug users don’t want other people to know where they are when they are away using … I am just being realistic, it’s alright for some, but there is others that could take advantage of it.” (LE)

Going further, LE and SP participants highlighted the capacity for mobile location data from apps to be misused by abusers and individuals attempting to target the vulnerable, with both groups agreeing that data availability should be restricted to trusted persons with limited opportunities to update arrangements.

“People that are vulnerable that are in abusive relationships (…) that scares me a bit. It shouldn’t be partners, or people like that, it has to be your next of kin and a peer support, nae boyfriends (…) you cannae add just anybody tae it.” (LE)

In contrast, critical considerations from FM participants focused on responder capacity, and, more fundamentally, how some PWUO lack trusted individuals who could function as OD responders

“This is dependent on finding suitable people as responders (…) some people are lucky enough, are in a fortunate position of having a number of people they can depend on, whereas other people are in a situation where they’ve got virtually no one to depend on and so the system falls down at that point.” (FM)

The importance of trust in alert responders was discussed at length in each focus group. This enabled participants to outline the characteristics desirable of first responders. Peer-support workers, and/or people in abstinence-based recovery were preferred by respondents.

“I definitely think like peer support is good. You dinnae want your family involved in things like that.” (LE)

“I think it should be people that are trained in naloxone that maybe are in recovery, and not actively using.” (SP)

The expressed confidence among participants towards peer-led approaches to harm reduction has clear implications for designers and organisations providing services for people who use drugs. Participants proposed that these concerns and preferences be considered at the design stage, by drawing on end-user input.

### Readiness—a double bind

The task of engaging high needs PWUO in digital interventions was discussed across participant groups and findings suggested willingness to participate constitutes a ‘readiness double bind’. That is, those at highest risk, and most in need of interventions, are the least able to engage with them, while individuals more likely to be receptive may not be interventions’ target population. This notion of ‘readiness’ effectively divided PWUO by the likelihood with which they would be willing to accept OD prevention interventions.

Perceived willingness to engage—closely linked to acceptability—was understood by LE and FM participants as a ‘recovery decision’, possibly reflective of a stage of change beyond that typifying certain ODART interventions’ target population.

“To be honest, if the addicts would use it, would initiate it [overdose monitoring ‘call’], I think it would work well. It’s just getting them to initiate it. So it would need to be them that changed and that’s just not going to happen.” (FM)

“How willing are they to admit they’re having a hit? I would think it’s a stage nearer to stopping.” (LE)

The irony of the double bind was noted across participant groups, with the pursuit of acceptable solutions for people at high risk of overdose understood as a perplexing issue. Essentially, the content under this theme highlights a chasm between proposed technical solutions and intervention uptake in the social realm.

“This sounds so derogatory, but if it was low down the scale in addiction, if they weren’t so addicted and they weren’t so out of their face kind of thing, yes, then that would be perfect. But higher scale of addiction, I just can’t see it being implemented from the start.” (FM)

Across focus groups, there was a general understanding that people seeking or in abstinence-based recovery, though often easier to engage, are not necessarily ODART interventions’ intended beneficiaries.

“Maybe more so for people who are supporting people who are actively using, or if people are in a recovery situation. But when they are in the height of the chaos of actively using, I am not so sure.” (SP)

However, the importance of peer buy-in to raise awareness among hardly reached PWUO was commonly discussed as a means to maximising intervention uptake.

“I think we are going to have a hard time with people who are like actively using. But just having the peers knowing what to do and them having it [OD responder app], that’s a start, I think.” (LE)

The double bind issue facing developers and implementation teams was well developed under this theme. Participants attempted to reconcile these challenges through solutions largely perceived to be found within the macroenvironmental dimension.

### Balancing technological sensitivity, harm reduction, safety and ambivalence

The analysis uncovered a number of interconnected challenges faced by developers and implementation teams related to creating acceptable products that can feasibly support fatal OD prevention. Under the meta theme ‘challenges to acceptability’, participant data describe how digital harm reduction interventions must strike a delicate balance between technological sensitivity, harm reduction and safety, amidst high levels of health ambivalence among the target population.

Understanding that severe withdrawal can follow naloxone administration, participants across groups noted how the sensitivity of naloxone-based technologies could determine their acceptability to PWUO. The socio-technical challenge this presents to designers and services working with PWUO was partially outlined by one LE participant, who, acknowledging personal risk, reflected on a recent using experience.

“I’m going to use, and I don’t want anyone to interrupt me and I don’t want to be around anybody, but at the same time (…) this is super high risk and I definitely don’t want to die but I know that I could.” (LE)

It was generally accepted that avoidance of fatal overdose supersedes any withdrawal experience precipitated by overdose reversal. However, in reference to an automatic OD response device (naloxone dispenser), another participant helped to further clarify the elusive ‘all or nothing’ boundary between a desired ‘high’ feeling and overdose, and the disruption and consequent upset that an unnecessary alert/response might cause.

“It might just set it off if you are just really high, and on the edge of going over so then you are going to be pissed off.” (LE)

In reference to the prototype automatic naloxone dispenser, one FM participant reasoned:

“I don’t see anybody consenting to that [naloxone implant]. I think you’d need to do that under duress.” (FM)

Consistent with this notion, and related to the theme of trust (above), one SP participant reflected on the issue of capacity in relation to OD prevention, before delineating their expectations that designers and implementation teams should adopt responsibility for ensuring clients are made aware of the implications of participating in interventions.

“I think the architecture to a system like this [naloxone responder app] has to almost assume a level of incapacity, not from the perspective of allowing the system to go and do whatever harm reduction is necessary, but to assume that people may not have awareness of this and to act accordingly.” (SP)

Though the relationship was underarticulated within the dataset, this issue of capacity/consent also appeared closely linked to ‘health ambivalence’—a phenomenon noted across focus groups to be characteristic of some PWUO, consistent with wider harm reduction literature [40].

“There was nae way I was even bothered about that and then when I couldn’t be arsed whether I lived or died, I wasn’t certainly pressing any buttons to let anybody ken I was taking drugs, I was just wanting the drugs in me to make sure I was feeling better, so I don’t think that would work at all.” (LE)

Under this eclectic theme, findings defined the unique socio-technical task of engaging and retaining PWUO in ODART interventions and bringing to the fore unspoken tensions between the principles of harm reduction and health ambivalence (otherwise characterisable as practicing the right not to engage with interventions). Participant data showed how these facets of the design challenge appear intricately connected to one another, with their correct balancing key to ‘solving’ the puzzle of acceptability.

## Discussion

### Principal findings

Willingness to support and/or participate in ODART interventions was high among participants. However, the findings presented here referred most immediately to the challenges arising from the fit between the proposed technical solutions, at risk populations and the contexts within which interventions are to be delivered. Trust in technology, systems and people was regarded as central to the acceptability and feasibility of digital OD prevention interventions for PWUO. Trust was further implicated through its relationship to autonomy and capacity, factors which participants felt developers and intervention implementation teams must balance with the principles of harm reduction and the need to keep people safe. However, although at high risk of OD due to intersecting health and social care needs, participants questioned the ‘readiness’ of some PWUO to engage with ODART, despite their standing the most to gain. Across groups, stakeholders agreed that multi-level buy-in would be essential to engaging those at risk and meaningfully reducing fatal OD. In response to the challenges identified, participants appealed to peer endorsement, and service provider support to promote the uptake and retention of PWUO in programmes.

### Integration with the current literature

Trust was shown to be central to ODART acceptability in this study, confirming perhaps the most universal finding in this space. Explicit discussions of trust were reported in several of the reviewed articles above [25, 26], while the concept was implicated in all but one of the remaining studies. Present findings align closely with those of Marcu et al [25] who likewise found trust to have referents across technology, people and systems (i.e. statutory services). From a technological perspective, naloxone responder networks were trusted to reduce fatal OD. Where Marcu and colleagues found interpersonal trust to promote harm reduction education, this work showed peers in or seeking recovery to be preferred OD responders. Additionally, present results reinforce and expand upon accounts of within-group distrust highlighted in the literature [25, 27], adding that individuals ‘in the throes’ of active drug use would be the least trusted OD responders. Distrust of systems, and particularly the police and statutory services, was also confirmed by this work.

Solutions to the readiness ‘double bind’ for high needs PWUO, who stand to benefit most from interventions [27] yet are unable to access them, are lacking. ODART, like other harm reduction interventions, demand a degree of client engagement if they are to be successful. However, the barriers identified above suggest that engagement with ODART entails comparatively greater engagement, than other options, and while the benefits are potentially considerable (e.g. prevention of death) they may seem more remote. In comparison, individuals utilising supervised consumption sites (SCS) are benefitting not just from monitored consumption, but also from the provision of a place to use around people in whom they have trust [41]. Take home naloxone (THN) has few, if any, engagement requirements and provision of the medication can be easily tacked on to existing interventions such as medication-assisted treatment and needle and syringe provision [12]. Drawing on this learning some PWUO might be engaged in ODART interventions through existing THN pathways, such as prior to release from prison or upon discharge from acute care and detoxification/residential rehabilitation services when opioid tolerance may be lower [22, 42–44]. Building upon Scottish digital inclusion work [22], present efforts also indicate that uptake of ODART among this population will necessarily involve peer champions and harm reduction-oriented organisations to promote awareness of, and trust in, interventions and will also require interventions to be co-designed appropriately for meeting end-users’ needs.

Present investigations identified a complex balancing of technological sensitivity, harm reduction and safety that must be achieved for digital OD interventions to be acceptable to PWUO. In essence, acceptable solutions are those which support autonomy around drug use, and operate unnoticed, with precision sensitivity, while being optimally accessible for individuals facing diverse and/or dynamic social circumstances. Consistent with earlier work [26, 27], false positives were shown to be particularly unacceptable to PWUO due to their potential to precipitate opioid withdrawal following naloxone intervention. Attention to the calibration of various biometrics is seen as crucial to improving the specificity of OD detection [26]. Yet, findings from an SCS in Canada demonstrate how OD response can be made more acceptable to PWUO, through titration of naloxone and supplementary oxygen to avoid severe withdrawal [45, 46]. However, while the minimisation of false positives might satisfy some PWUO to engage with ODART, pervasive health ambivalence presents a far greater challenge to the feasibility of digital OD prevention strategies. For present participants, such ambivalence, amid contexts of severe harm and death called into question the nature of capacity and consent. This tension between harm reduction and safety demands greater attention than it has so far received in the ODART literature.

### TPOM-ODART framework

The TPOM framework [28] was selected for its capacity to accommodate stakeholder insights into the socio-technical task digital OD prevention presents to developers, implementation teams and supporters. As anticipated, the four broad dimensions of the TPOM were sufficient to help categorise data and support the visualisation of the interrelations between dimensions. However, while the emergent themes are in partial alignment with the referent model (see Additional file 1: Appendix 1), their discussion here supports the case to augment the model to better fit the population/proposed technological goal. Thus, spanning the technological and social/human dimensions of the TPOM-ODART (see Figure 1) ‘readiness—a double bind’ and ‘balancing harm reduction, safety and ambivalence’ speak to socio-technical challenges highly specific to PWUO.

Balancing harm reduction, safety and ambivalence’ reflects the complex task of harmonising PWUO were described, through the living experience of participants, as seeking solutions which are so responsive and accurate (technological performance heuristic) that they can differentiate between a desirable level of heavy sedation and OD, alerting third parties only in instances of the latter. This desired borderline level of intoxication may be a norm among PWUO (attitudes and expectations heuristic), and particularly those at highest risk, for whom intervention acceptability may hinge on a single false positive.

The new theme ‘readiness—a double bind’ appears to sit well within the ‘people’ dimension of the TPOM, yet the heuristic social/human factors derived by Cresswell et al [28] seem insufficient to accurately capture this pervasive feature of problem drug use [47]. The original model factors of ‘engagement’ and ‘user input in design’ may speak to the maximisation of uptake of digital harm reduction interventions, yet for individuals with the highest needs, the right not to engage in treatment must be respected even in cases where severe risk to health is likely and capacity to consent either way is questionable. Once more reflecting the interrelated nature of the socio-technical challenge, this theme is closely linked to the technological factor of performance (e.g. to what extent do ODART interventions support OD prevention among high risk PWUO?) and so could plausibly be depicted as spanning both dimensions. The decision to situate this theme squarely in the social/human dimension, however, was determined on the merits of this being a distinctive feature of the population.

### Strengths and limitations

This work is timely and coincides with the urgent impetus to respond to DRD crises and significant political appetite, as well as the enhanced availability of digital healthcare funding since COVID-19 [48, 49]. To the knowledge of the authors, this is the first study to attempt to combine primary data regarding the acceptability of technology for DRD prevention from the perspective of PWUO, affected family members and service providers. The specific strengths of this study lie in those areas which were identified as complementing the academic literature. For example, the themes presented here were intentionally selected for their capacity to elucidate the socio-technical task faced by technologists and intervention implementation teams. The subtleties of the challenge such as the delicate balances required to achieve acceptability may be highly specific to this population. Controversial issues relating to capacity and consent, as well as challenges concerning populations who may remain out of reach, despite programmes’ best efforts, were also brought to the fore. The opportunity presented to support the development of the TPOM, and socio-technical theory more broadly was also welcomed.

The pervasive experience of digital exclusion among PWUO [22] likely limited the participant pool, and thus, the generalisability of any findings. For example, smartphone ownership and connectivity were 100% among LE participants, in stark contrast to estimates across the literature [22, 26]. Participation from individuals and groups involved in DRD prevention at the level of policy, and elected officials would have added finer detail concerning the macroenvironment, and thus, greater strategic insight into the overall feasibility of digital programmes. More fundamentally, hypothetical/perceived acceptability of ODART may be at odds with actual rates of usage. Thus, the integration of such devices will need to be trialled in real world settings to better understand acceptability/feasibility.

### Relevance for policy and practice

Findings from this work could be used to help shape and improve the challenges present for ODART developers. Services considering the adoption of existing technologies, or developing apps, should be sensitive to these findings to maximise utility. Service users and using populations should be at the forefront of any innovative exercise so that ideas are not informed by ground truth but also allow informative dialogue to maximise their effective use.

## Conclusions

Effective digital OD prevention strategies will need to be sensitive to the complex relationships between technological, social/human, organisational and wider macroenvironmental factors which can enable or impede intervention delivery. An augmented TPOM framework called the TPOM-ODART was developed to support this area. Concerns of trust, readiness, accessibility, capacity and the necessity for meaningful collaboration are factors central to technology acceptability for PWUO.

## Supplementary Information

**Additional file 1. Appendix 1**. Focus group procedure: presentation slides.

**Additional file 2. Appendix 2: Table 1**. The Technology, People, Organizations, and Macroenvironmental factors (TPOM) framework, with example descriptions of dimensions [28].

## Data Availability

The data that support the findings of this study are available on request from the corresponding author [JD]. Transcripts/audio files are not publicly available due to them containing information that could compromise participants’ privacy/consent.

## Abbreviations

DRD: Drug-related death
OD: Overdose
ODART: Overdose alert and response technologies
PWUO: People who use opioids
TPOM: Technology, People, Organisations, Macroenvironment framework
LE: Lived experience of problem opioid use
FM: Family members
SP: Service providers

## References

[1] United Nations Office on Drugs and Crime. World Drug Report 20212021. Available from: https://www.unodc.org/unodc/en/data-and-analysis/wdr2021.html.

[2] Ahmad F, Rossen L, Sutton P. Provisional drug overdose death counts: 2021. National Center for Health Statistics. 2022; 2022(14–5–2022). Available from: https://www.cdc.gov/nchs/pressroom/nchs_press_releases/2021/20211117.htm#:~:text=Provisional%20data%20from%20CDC's%20National,same%20period%20the%20year%20before.

[3] National Records of Scotland. Drug-related Deaths in Scotland in 2020. 2021. Available from: https://www.nrscotland.gov.uk/statistics-and-data/statistics/statistics-by-theme/vital-events/deaths/drug-related-deaths-in-scotland/2020.

[4] Barocas JA, Wang J, Marshall BD, LaRochelle MR, Bettano A, Bernson D (2019). Sociodemographic factors and social determinants associated with toxicology confirmed polysubstance opioid-related deaths. Drug Alcohol Dep.

[5] European Monitoring Centre for Drugs and Drug Addiction. Action framework for developing and implementing health and social responses to drug problems. 2021. Available from: https://www.emcdda.europa.eu/publications/health-and-social-responses-a-european-guide_en.

[6] Public Health Scotland. The national drug-related deaths database (Scotland) report: analysis of deaths occurring in 2017 and 2018. 2022. Available from: https://publichealthscotland.scot/media/16202/2022-07-26-ndrdd-report_revised_v1.pdf.

[7] Pizzicato LN, Drake R, Domer-Shank R, Johnson CC, Viner KM (2018). Beyond the walls: risk factors for overdose mortality following release from the Philadelphia Department of Prisons. Drug Alcohol Dep.

[8] Aldridge RW, Story A, Hwang SW, Nordentoft M, Luchenski SA, Hartwell G (2018). Morbidity and mortality in homeless individuals, prisoners, sex workers, and individuals with substance use disorders in high-income countries: a systematic review and meta-analysis. Lancet.

[9] Strang J, Powis B, Best D, Vingoe L, Griffiths P, Taylor C (1999). Preventing opiate overdose fatalities with take-home naloxone: pre-launch study of possible impact and acceptability. Addiction.

[10] Moustaqim-Barrette A, Elton-Marshall T, Leece P, Morissette C, Rittenbach K, Buxton JA. Environmental scan naloxone access and distribution in Canada. 2019. Available from: https://substanceuse.ca/sites/default/files/2021-03/CRISM_Enviro-Scan_Final-Draft_June18.pdf.

[11] Robinson A, Christensen A, Bacon S (2019). From the CDC: the Prevention for States program: preventing opioid overdose through evidence-based intervention and innovation. J Saf Res.

[12] Public Health Scotland. National naloxone programme Scotland monitoring report 2019/20 & 2020/21. 2022. Available from: https://publichealthscotland.scot/media/12949/22-05-03-naloxone-report.pdf.

[13] Chen Y, Wang Y, Nielsen S, Kuhn L, Lam T (2020). A systematic review of opioid overdose interventions delivered within emergency departments. Drug Alcohol Depend.

[14] Scottish Families Affected by Alcohol and Drugs. Take-home naloxone. 2021. Available from: https://www.sfad.org.uk/support-services/take-home-naloxone.

[15] Barnsdale L, Gounari X, Graham L. The national drug related deaths database (Scotland) report. Analysis of deaths occurring in 2015 and 20162018. Available from: https://www.isdscotland.org/Health-Topics/Drugs-and-Alcohol-Misuse/Publications/2018-06-12/2018-06-12-NDRDD-Report.pdf.

[16] Papamihali K YM, Graham B, Karamouzian M, Slaunwhite AK, Tsang V, Young S, Buxton JA. Convenience and comfort: reasons reported for using drugs alone among clients of harm reduction sites in British Columbia, Canada. Harm Reduct J. 2020. Available from: https://web.archive.org/web/20201126225034id_/https://harmreductionjournal.biomedcentral.com/track/pdf/; 10.1186/s12954-020-00436-6.pdf. 10.1186/s12954-020-00436-6 PMC7682134 33228676

[17] Public Health Ontario. Overdose in Canada: an epidemic within a pandemic. 2021. Available from: https://www.publichealthontario.ca/en/About/News/2021/Overdose-in-Canada.

[18] Dorsey ER, Topol EJ (2016). State of telehealth. New Engl J Med.

[19] Oteo A, Daneshvar H, Baldacchino A, Matheson C (2023). Overdose alert and response technologies: state-of-the-art review. J Med Internet Res.

[20] Mark TL, Treiman K, Padwa H, Henretty K, Tzeng J, Gilbert M (2022). Addiction treatment and telehealth: review of efficacy and provider insights during the COVID-19 pandemic. Psychiatr Serv.

[21] Teck JT, Zlatkute G, Perez A, Dritschel H, Ghosh A, Potenza MN, Ambekar A, Ekhtiari H, Stein D, Khazaal Y, Arunogiri S (2023). Key implementation factors in telemedicine-delivered medications for opioid use disorder: a scoping review informed by normalisation process theory. Lancet Psychiatry.

[22] Matheson C, Carver H, Parkes T, Daneshvar H, Schofield J, Dumbrell J, et al. Digital inclusion to prevent drug related deaths: Scoping user needs. Drugs Res Netw Scotl. 2022. Available from: https://dspace.stir.ac.uk/retrieve/0c1531e5-7003-4344-9456-8b7eb6d90d66/Scoping%20user%20needs%20-%20report%20-%20version%204%20final%20200122.pdf.

[23] Ahamad K, Dong H, Johnson C, Hyashi K, DeBeck K, Milloy MJ, Wood E (2019). Factors associated with willingness to wear an electronic overdose detection device. Addict Sci Clin Pract.

[24] Bardwell G, Fleming T, McNeil R, Boyd J (2021). Women's multiple uses of an overdose prevention technology to mitigate risks and harms within a supportive housing environment: a qualitative study. BMC Womens Health.

[25] Marcu G, Aizen R, Roth AM, Lankenau S, Schwartz DG (2020). Acceptability of smartphone applications for facilitating layperson naloxone administration during opioid overdoses. Jamia Open.

[26] Tsang VWL, Papamihali K, Crabtree A, Buxton JA (2021). Acceptability of technological solutions for overdose monitoring: perspectives of people who use drugs. Subst Abuse.

[27] Kanter K, Gallagher R, Eweje F, Lee A, Gordon D, Landy S (2021). Willingness to use a wearable device capable of detecting and reversing overdose among people who use opioids in Philadelphia. Harm Reduct J.

[28] Cresswell K, Williams R, Sheikh A (2020). Developing and applying a formative evaluation framework for health information technology implementations: qualitative investigation. J Med Internet Res.

[29] Gentles SJ, Charles C, Ploeg J, McKibbon KA (2015). Sampling in qualitative research: insights from an overview of the methods literature. Qual Rep.

[30] Ritchie J, Lewis J, Elam G. Designing and selecting samples. Qual Res Methods. 2003:77–108.

[31] Kitzinger J (1994). The methodology of focus groups: the importance of interaction between research participants. Sociol Health Illn.

[32] Rhodes T (2002). The ‘risk environment’: a framework for understanding and reducing drug-related harm. Int J Drug Policy.

[33] Duff C (2009). The drifting city: the role of affect and repair in the development of “Enabling Environments”. Int J Drug Policy.

[34] Davis FD (1989). Perceived usefulness, perceived ease of use, and user acceptance of information technology.

[35] Latour B. Reassembling the social: an introduction to actor-network-theory2007. Available from: http://objects.avant.org/incredible-machines/Latour_Reassembling.pdf.

[36] Braun V, Clarke V (2006). Using thematic analysis in psychology. Qual Res Psychol.

[37] Braun V, Clarke V. Thematic analysis. 2012. Available from: 10.1037/13620-004.

[38] International Q. NVivo Qualitative Data Analysis Software. 1999. Available from: https://qsrinternational.com/nvivo/nvivo-products/.

[39] Sittig DF, Singh H. A new socio-technical model for studying health information technology in complex adaptive healthcare systems. Cognitive informatics for biomedicine: Springer; 2015. p. 59-80. Available from: 10.1136%2Fqshc.2010.042085 10.1136/qshc.2010.042085 PMC3120130 20959322

[40] Moore D (2004). Governing street-based injecting drug users: a critique of heroin overdose prevention in Australia. Soc Sci Med.

[41] Parkes T, Price T, Foster R, Trayner K, Sumnall HR, Livingston W (2022). ‘Why would we not want to keep everybody safe?’The views of family members of people who use drugs on the implementation of drug consumption rooms in Scotland. Harm Reduct J.

[42] Horton M, McDonald R, Green TC, Nielsen S, Strang J, Degenhardt L (2017). A mapping review of take-home naloxone for people released from correctional settings. Int J Drug Policy.

[43] Kestler A, Buxton J, Meckling G, Giesler A, Lee M, Fuller K (2017). Factors associated with participation in an emergency department–based take-home naloxone program for at-risk opioid users. Ann Emerg Med.

[44] Pearce LA, Mathany L, Rothon D, Kuo M, Buxton JA (2019). An evaluation of Take Home Naloxone program implementation in British Columbian correctional facilities. Int J Prison Health.

[45] Farrugia A, Neale J, Dwyer R, Fomiatti R, Fraser S, Strang J (2020). Conflict and communication: managing the multiple affordances of take-home naloxone administration events in Australia. Addict Res Theory.

[46] Watson TM, Kolla G, van der Meulen E, Dodd Z (2020). Critical studies of harm reduction: overdose response in uncertain political times. Int J Drug Policy.

[47] DiClemente CC, Schlundt D, Gemmell L (2004). Readiness and stages of change in addiction treatment. Am J Addict.

[48] Doraiswamy S, Abraham A, Mamtani R, Cheema S (2020). Use of telehealth during the COVID-19 pandemic: scoping review. J Med Internet Res.

[49] Jones CM, Shoff C, Hodges K, Blanco C, Losby JL, Ling SM (2022). Receipt of telehealth services, receipt and retention of medications for opioid use disorder, and medically treated overdose among medicare beneficiaries before and during the COVID-19 pandemic. JAMA Psychiat.

