# Supplementary material for: The acceptability of overdose alert and response technologies: introducing the TPOM-ODART framework
Source: Harm Reduct J. 2023 Mar 26;20:40. doi: 10.1186/s12954-023-00763-4 (PMC10040083; doi:10.1186/s12954-023-00763-4)
Supplement: Supplementary file 1 — Additional file 1. Appendix 1. Focus group procedure: presentation slides. [file 12954_2023_763_MOESM1_ESM.pdf]

Focus group procedure

The research team delivered a presentation to all focus group attendees describing the types of applications and devices in production/use in the ODART space before opening up for discussion.

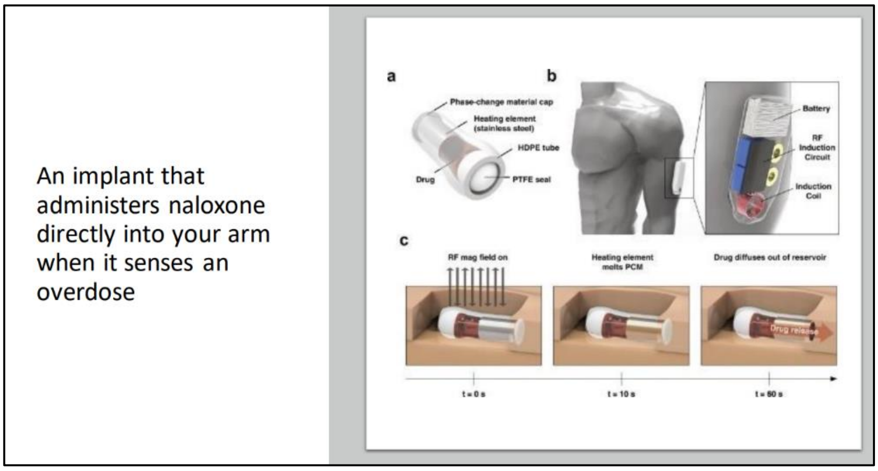

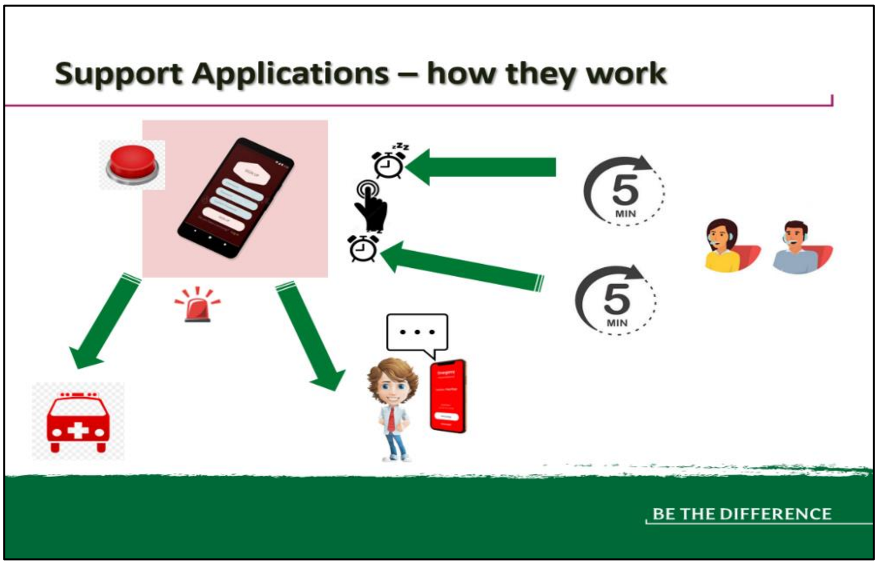

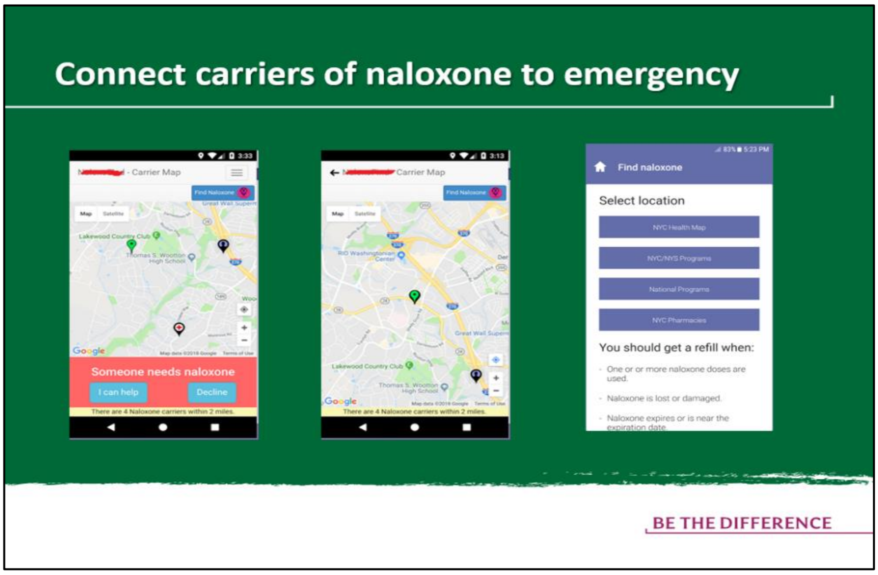

Oteo A, Daneshvar H. Focus group presentation slides [selection]. 2022.
