# Supplementary material for: The acceptability of overdose alert and response technologies: introducing the TPOM-ODART framework
Source: Harm Reduct J. 2023 Mar 26;20:40. doi: 10.1186/s12954-023-00763-4 (PMC10040083; doi:10.1186/s12954-023-00763-4)
Supplement: Supplementary file 2 — Additional file 2. Appendix 2: Table 1. The Technology, People, Organizations, and Macroenvironmental factors (TPOM) framework, with example descriptions of dimensions [28]. [file 12954_2023_763_MOESM2_ESM.pdf]

Table 1. The Technology, People, Organizations, and Macroenvironmental factors (TPOM) framework, with example descriptions of dimensions (Cresswell et al., 2020).

Factor and dimension	Description
	**Technological factors**
Usability	What is the ease of use and learnability of the technology?
Performance	Does the technology function as intended by developers?
Adaptability and flexibility	Can system design be changed to suit emerging needs?
Dependability	Is the system reliable and stable?
Data availability, integrity, and confidentiality	Is data in the system available, accessible, and usable for those who need it?
Data accuracy	Is the data in the system accurate?
Sustainability	Is use of the technology sustainable?
Security	Is the system secure?
	**Social/human factors**
User satisfaction	Who are the users? Are users satisfied with the technology?
Complete/correct use	Are features and functionality implemented and used as intended?
Attitudes and expectations	What benefits do users expect from using the technology and how can these be measured?
Engagement	Are users actively engaged in implementation, adoption, and optimization?
Experiences	Do users have negative experience with previous technologies?
Workload/benefits	Are the benefits and efforts relatively equal for all stakeholders?
Work processes	Does the system change relationships with patients, patterns of communication, and professional responsibilities (eg, increase of administrative tasks)?
User input in design	Is there effective communication between designers, information technology staff, and end users, as well as between management and end users?
	**Organizational context**
Leadership and management	Are management structures to support the implementation adequate?
Communication	Are aims, timelines, and strategy communicated?
Timelines	Are implementation timelines adequate?
Vision	What benefits do organizations expect from implementing the technology and how can these be measured? Is a coherent and realistic vision driving developments?
Training and support	Is the training adequate and realistic?
Champions	Are champions and boundary spanners utilized?
Resources	Is implementation adequately resourced? (includes technology, change management, and maintenance)
Monitoring and optimization	Is system performance and use monitored and optimized over time? Are lessons learned captured and incorporated in future efforts?
	**Wider macroenvironment**
Media	How is the technology viewed by the media and by the public? How does the organization view/manage media relations?
Professional groups	How is the technology viewed by professional groups?
Political context	What benefits do policymakers expect from the technology and how can these be measured? What is the national approach to achieving interoperability and does the system align with this? Is there a coherent vision, consistent approach, and a clear direction of travel, allowing a degree of local input?
Economic considerations and incentives	Are there clear incentives for organizations and users to implement? (eg, improvements in quality of care) Is sufficient funding in place to support the initiative?
Legal and regulatory aspects	Have legal and regulatory frameworks been established?
Vendors	Is vendor management effectively organized?
Measuring impact	Are various stakeholders working together to define, validate, test, and refine outcome measures and measurement strategies? Are outcome measures important, clinically acceptable, transparent, feasible, and usable?
